# Molecular mechanisms involved in the non-monotonic effect of bisphenol-a on Ca^2+^ entry in mouse pancreatic β-cells

**DOI:** 10.1038/s41598-017-11995-3

**Published:** 2017-09-18

**Authors:** Sabrina Villar-Pazos, Juan Martinez-Pinna, Manuel Castellano-Muñoz, Paloma Alonso-Magdalena, Laura Marroqui, Ivan Quesada, Jan-Ake Gustafsson, Angel Nadal

**Affiliations:** 1 CIBER de Diabetes y Enfermedades Metabólicas Asociadas (CIBERDEM) and Institute of Bioenginering, Miguel Hernández University of Elche, Elche, Alicante Spain; 2 Departamento de Fisiología, Genética y Microbiología, Universidad de Alicante, Alicante, Spain; 3 Department of Cell Biology and Biochemistry, Center for Nuclear Receptors and Cell Signaling, University of Houston, Houston, Texas USA; 4 Department of Biosciences and Nutrition, Karolinska Institut, Huddinge, Sweden

## Abstract

In regulatory toxicology, the dose-response relationship is a key element towards fulfilling safety assessments and satisfying regulatory authorities. Conventionally, the larger the dose, the greater the response, following the dogma “the dose makes the poison”. Many endocrine disrupting chemicals, including bisphenol-A (BPA), induce non-monotonic dose response (NMDR) relationships, which are unconventional and have tremendous implications in risk assessment. Although several molecular mechanisms have been proposed to explain NMDR relationships, they are largely undemonstrated. Using mouse pancreatic β-cells from wild-type and oestrogen receptor ERβ−/− mice, we found that exposure to increasing doses of BPA affected Ca^2+^ entry in an NMDR manner. Low doses decreased plasma membrane Ca^2+^ currents after downregulation of Cav2.3 ion channel expression, in a process involving ERβ. High doses decreased Ca^2+^ currents through an ERβ-mediated mechanism and simultaneously increased Ca^2+^ currents via oestrogen receptor ERα. The outcome of both molecular mechanisms explains the NMDR relationship between BPA and Ca^2+^ entry in β-cells.

## Introduction

Endocrine disrupting chemicals (EDCs) are defined by The Endocrine Society as “chemicals, or mixtures of chemicals, that interfere with any aspect of hormone action”^1^. Bisphenol-A (BPA) is a manmade chemical that forms the base component of the polycarbonate plastic used to produce epoxy resins and as a plasticizer in the manufacturing of other plastics such as PVC^2^. BPA is classified as an EDC, is found in the urine of 93% of USA citizens^3^ and has been associated with different non-communicable diseases including metabolic disorders^4,5^. It has been suggested that BPA may be involved in the aetiology of type 2 diabetes mellitus (T2DM) because it causes insulin resistance and disrupts pancreatic β-cell function in mice^6–8^. BPA acts at doses that are considered low^9^, ranging from 100 pM-1 nM in studies performed *in vitro*^10,11^ and after exposures ranging from 25 ng/kg/day to 100 µg/kg/day in animal models^12–15^. These doses are within the range of human exposure and below those used in traditional toxicological studies^16,17^. Notably, industry workers manufacturing, or using, BPA have approximately 70 times higher BPA levels in urine compared to adults^18^.

BPA affects the pancreatic β-cell insulin content and secretion via oestrogen receptors ERα and ERβ actions outside the nucleus. ERα increases pancreatic β-cell insulin gene expression and, therefore, insulin content^19^, whereas ERβ blocks K_ATP_ channels with a subsequent rapid increase in insulin release^20^. It is of note that in the latter case, the relationship between the BPA dose and effect occurs in a monotonic dose-response (MDR) manner^20^, while in the former, this relationship occurs in a non-monotonic dose-response (NMDR)-dependent manner^19^. MDR curves present linear or nonlinear relationships whose slopes are either positive or negative. In contrast, NMDRs occur when the slope of the curve changes direction at some point within the range of the doses examined^17^. Exposure to EDCs usually elicits NMDR curves, a phenomenon particularly common in the case of BPA^21^. NMDR relationships obtained with EDCs have important implications for risk assessment because safety at high doses does not guarantee that low doses are safe. Therefore, the concept of potency, highly used in risk assessment toxicology, cannot be applied, making it difficult or even impossible to establish a threshold below which no observable effect occurs. Despite the important implications that this phenomenon presents for the toxicological sciences, the molecular mechanisms underlying the NMDRs for EDCs remain poorly understood.

Pancreatic β-cells, similar to neurons and muscle fibres, are excitable cells, and therefore, they generate electrical signals that couple stimuli to secretion. The electrical activity of a cell is defined as the ability to conduct, transmit and receive electrical signals that result from the opening and closing of specific ion-channel proteins in the plasma membrane. Pancreatic β-cells produce a particular pattern of electrical activity in bursts of action potentials in response to high extracellular glucose levels^22^. When extracellular glucose is low, the resting membrane potential of the β-cell is maintained at approximately −70 mV by a particular type of ion channel, namely, ATP-sensitive K^+^ channels (K_ATP_). These channels close when glucose is metabolized, depolarizing the plasma membrane and triggering the opening of several types of voltage-gated channels, which leads to the generation of action potential bursting^23^. As a consequence, the intracellular Ca^2+^ concentration ([Ca^2+^]_i_) oscillates^24^, generating a signal that triggers the exocytosis of insulin granules^23^. Based on the importance of ion channels in the physiology of pancreatic β-cells, it has been proposed that the defective insulin secretion that occurs in T2DM is the result of inadequate β-cell electrical activity in response to secretagogues^25^.

In the case of mouse β-cells, the depolarizing phase of the action potential involves exclusively voltage-gated Ca^2+^ channels, while the repolarizing phase implicates different types of K^+^ channels^22^. There are three subfamilies of voltage-gated Ca^2+^ channels: (1) L-type Ca^2+^ channels that are inhibited by dihydropyridines (DHPs) that comprise the Cav1.1-4 channels; (2) the Cav2.1 or P/Q-type channels, which are sensitive to ω-agatoxin, the Cav2.2 or N-type channels, which are sensitive to ω-conotoxin, and the Cav2.3 or R-Type channels, which are sensitive to SNX482; and (3) the T-Type Ca^2+^ channels comprising Cav 3.1–3^26^. In mouse β-cells, 50–60% of the total Ca^2+^ current flows through L-type Cav1.2 channels, which are key players in the insulin release process^27,28^. The remaining 40–50% of the Ca^2+^-current is equally divided between P/Q-type Ca2.1, N-Type Cav2.2 and R-type-Cav2.3^28^. Islets from Cav2.3−/− mice show that the R-type channels are involved in the second phase of insulin release^29^.

Here, we show that exposure of pancreatic β-cells to BPA (from 100 pM to 1 µM) decreased Ca^2+^ entry via an ERβ-dependent pathway that involves the transcriptional regulation of Cav2.3 channels. Conversely, ERα increased Ca^2+^ entry only in response to higher BPA concentrations (100 nM and 1 µM) after increasing Ca^2+^ currents in a pathway involving phosphoinositide 3-kinase (PI3K). A low dose of 1 nM activated only the ERβ-dependent pathway, decreasing Ca^2+^ entry. Higher doses of BPA at 100 nM and 1 µM simultaneously activated both the ERα and ERβ-dependent pathways, which counteract each other. The combined opposing effects of ERα and ERβ upon Ca^2+^ entry mechanistically explain the NMDR relationship observed for BPA actions.

## Results

### BPA reduced the amplitude of the action potentials in pancreatic β-cells

**Figure 1:**
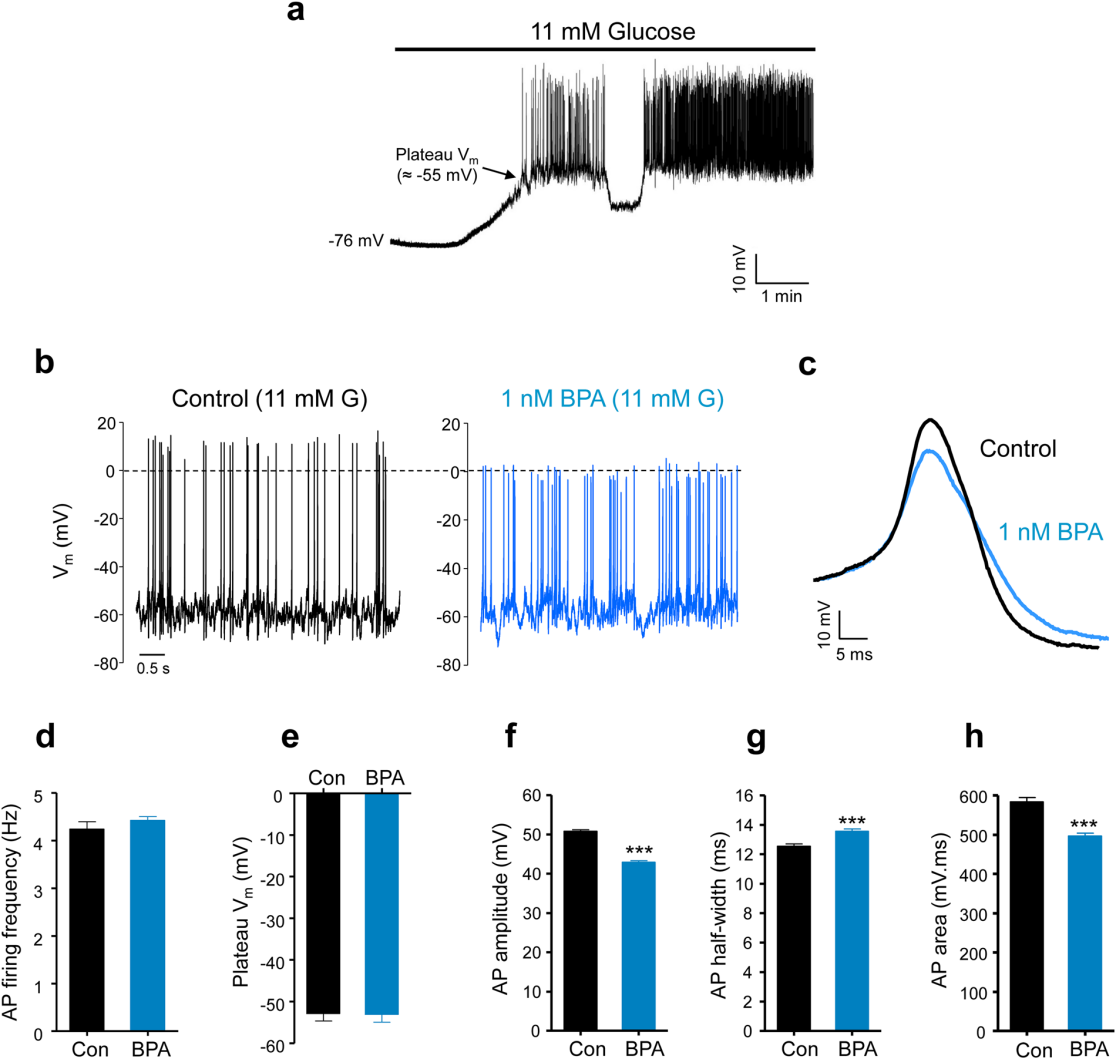
Effect of BPA on the action potentials of mouse pancreatic β-cells. (**a**) Typical depolarization and action potential firing in bursts in response to a stimulatory glucose level (11 mM) from 0 mM glucose, recorded using the perforated-patch clamp technique at 36 °C. The resting membrane potential at 0 mM glucose and the plateau membrane potential from which the firing of the action potentials was initiated at 11 mM glucose are indicated. (**b**) Action potential firing during a burst at 11 mM glucose in the presence of vehicle (*Control*, black panel) or 1 nM BPA (blue panel). The dashed line indicates 0 mV. (**c**) Superimposed representative action potentials at 11 mM glucose in the control and 1 nM BPA. (**d**) Average action potential firing frequency at 11 mM glucose in the control (1589 action potentials from *n* = 11 cells) and 1 nM BPA (1780, *n* = 11 cells). (**e**) Average plateau membrane potential at 11 mM glucose in control (*n* = 11) and 1 nM BPA (*n* = 11). (**f**) Average action potential amplitude at 11 mM glucose from plateau membrane potential to peak (1600 and 1780 action potentials in the control and 1 nM BPA, respectively; *n* = 11). (**g**) Average action potential duration at half of the amplitude at 11 mM glucose (1600 and 1780 action potentials in the control and 1 nM BPA, respectively; *n* = 11). (**h**) Average area under the action potential at 11 mM glucose (1600 and 1780 action potentials in the control and 1 nM BPA, respectively; *n* = 11). Data are represented as the mean ± s.e.m.; Student’s *t*-test: ***P < 0.001.

**Figure 2:**
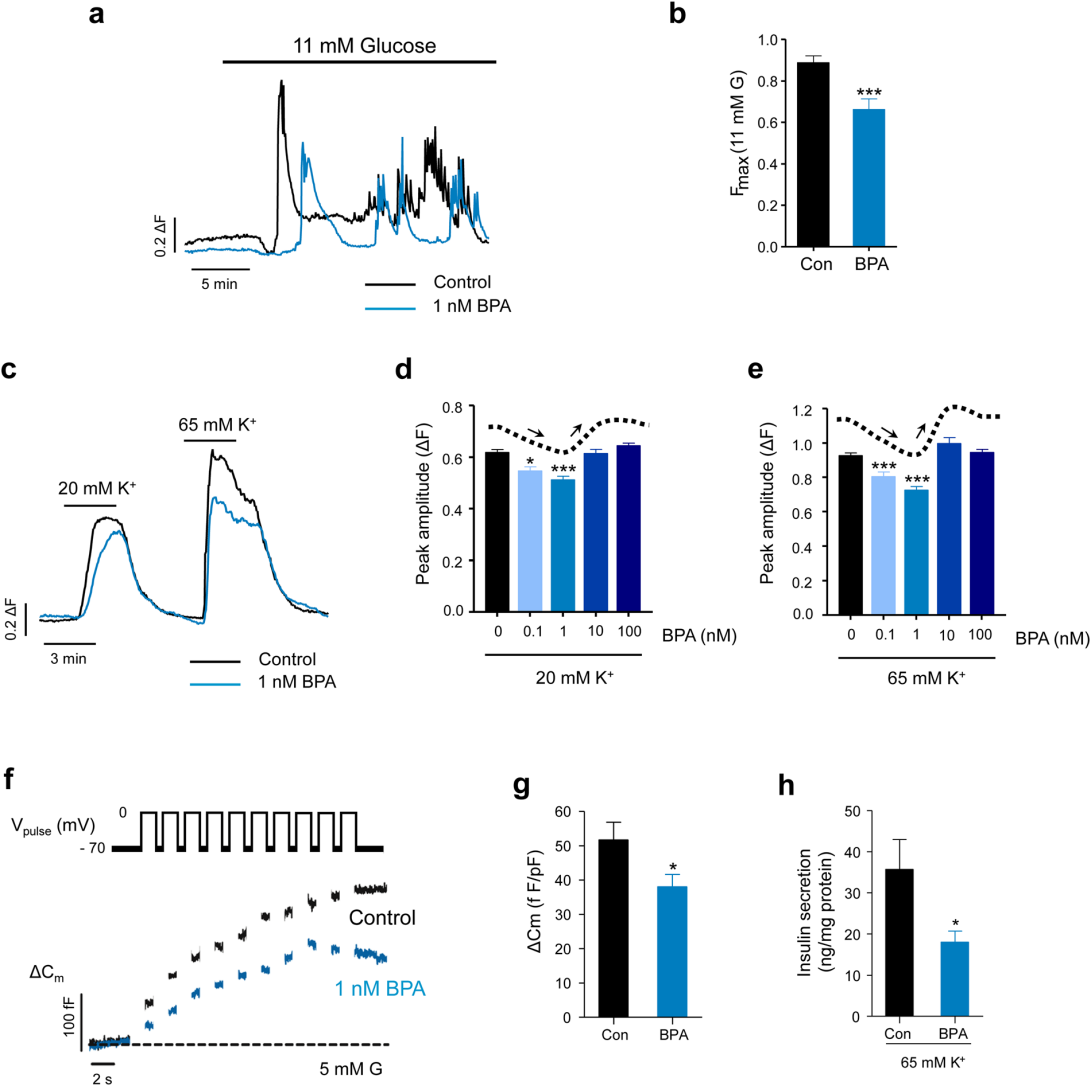
BPA inhibits Ca^2+^ entry, exocytosis and insulin secretion in mouse pancreatic β-cells. (**a**) Representative superimposed recordings of fura-2 Ca^2+^ fluorescence of isolated islet cells in response to a stimulatory glucose level (11 mM) in the control (black trace) and 1 nM BPA (blue trace). (**b**) Quantification of average amplitude change measured in (**a**). Measurements from 38 (black) and 21 (blue) cells from 2 independent experiments. (**c**) Representative superimposed recordings of fura-2 Ca^2+^ fluorescence of isolated islet cells in response to 20 and 65 mM KCl in the control (black) and 1 nM BPA (blue) when 100 μM diazoxide was present in the bath solution. (**d**) Average amplitude of the intracellular Ca^2+^ responses elicited by 20 mM KCl in cells exposed to increasing doses of BPA for 48 hours (*n* = 139, 83, 145, 66 and 78 cells, obtained from 4 independent experiments). The dotted line and arrows depict the non-monotonic dose-response relationship at the top of the histogram. (**e**) Same as in (**d**) but with 65 mM KCl (*n* = 139, 68, 143, 66 and 78 cells, obtained from 4 independent experiments). (**f**) Representative superimposed recordings of the membrane capacitance increase (lower panel) in response to depolarizing voltage steps (−70 to 0 mV, 500 ms duration; upper panel) in isolated β-cells in control (black) and 1 nM BPA (blue) at 5 mM glucose. (**g**) Quantification of average increase in capacitance (normalized to the cell size in pF) at the 10^th^ voltage pulse of the experiment shown in (**f**) (control: *n* = 20 cells; BPA: *n* = 21 cells; obtained from 6 independent experiments). (**h**) Average increase in insulin secretion of islets (normalized to the islet protein content in mg) induced by extracellular application of 65 mM KCl (n = 10–11 groups of 5 islets per condition from 8 animals). Data are represented as the mean ± s.e.m. In **b**,**g**,**h:** Student’s *t*-test: *P ≤ 0.05; ***P < 0.001. In **d**,**e**: One-way ANOVA followed by Dunnett’s post hoc test, *P ≤ 0.05; ***P < 0.001 vs. control.

To investigate the action of BPA on pancreatic β-cell signalling, we recorded electrical activity from isolated β-cells in culture using the perforated patch-clamp technique. The membrane potential of the β-cells remained stable at approximately −70 mV in the absence of a stimulatory glucose concentration. When we added a stimulatory glucose concentration (11 mM), the membrane potential rapidly depolarized to a plateau at approximately −50 mV, where Ca^2+^ action potentials were generated (Fig. 1a). Using single β-cells cultured for 48 hours in the presence of either the vehicle or BPA, we found that BPA did not affect either the glucose-induced membrane potential plateau in the presence of 11 mM glucose (Fig. 1b,d) or the frequency of the action potential firing (Fig. 1b,e). However, BPA modified the waveform of these action potentials, reducing the amplitude (Fig. 1b,f) and increasing the duration at half the amplitude (Fig. 1c,g). Importantly, the area under the action potential was also reduced (Fig. 1c,h), suggesting a decrease in the amount of Ca^2+^ entering the cell during each action potential. Because the bursting pattern of the electrical activity in whole islets of Langerhans generates intracellular Ca^2+^ oscillations, we assessed the increase in [Ca^2+^]_i_ in response to 11 mM glucose in islets cultured in the absence or presence of 1 nM BPA for 48 hours. Isolated islet cells treated with BPA showed a diminished Ca^2+^ entry, manifested as a smaller first peak size induced by glucose (Fig. 2a,b) and a decreased area under the curve during the oscillatory phase in whole islets (Supplementary Fig. 1).

### BPA decreased voltage-induced calcium entry in an NMDR manner

To determine whether Ca^2+^ entry was altered independently of glucose actions, we depolarized cells using a high extracellular K^+^ pulse in a non-stimulatory glucose concentration of 3 mM and in the presence of a K_ATP_ opener diazoxide (100 µM)^30^. Two concentrations of high KCl were used (20 mM and 65 mM), which produced depolarizations close to the threshold (E_K_ ≈ −45 mV) or peak (E_K_ ≈ −15 mV) of the depolarization-induced Ca^2+^ entry. Under these conditions, BPA-treated cells showed a reduced depolarization-induced Ca^2+^ entry (Fig. 2c). This protocol was useful for two reasons. First, it indicated that Ca^2+^ entry through voltage-gated Ca^2+^ channels must be decreased (see later). Second, it allowed us to easily and rapidly test the effect of different BPA concentrations on Ca^2+^ entry. These experiments showed a remarkable phenomenon: the amplitude of the Ca^2+^ signal induced by both 20 mM and 65 mM extracellular K^+^ was reduced in an NMDR manner. Low concentrations (100 pM and 1 nM BPA) decreased Ca^2+^ entry compared to the control, whereas higher concentrations (10 nM and 100 nM BPA) had no effect (Fig. 2d,e).

### The BPA treatment affected exocytosis and insulin release

To study the physiological consequences of reduced Ca^2+^ entry in the exocytosis of insulin granules, we measured the capacitance of the plasma membrane in response to depolarizing square pulses (−70 to 0 mV, 500 ms duration) using electrophysiological methods. Cells exposed to 1 nM BPA showed decreased exocytosis compared to the control in the presence of 5 mM glucose (Fig. 2f,g). This reduction was observed in the total exocytosis (Fig. 2f,g) and the initial release (first pulse, readily releasable pool) (data not shown). Similarly, BPA reduced insulin release in response to depolarizing extracellular K^+^ at low glucose (Fig. 2h). The effect of BPA on high glucose-induced insulin secretion was the opposite: BPA potentiated glucose-induced insulin release and augmented the insulin content (Supplementary Fig. 1), as previously described^6,19^. These results point to dual regulation of exocytosis by BPA, which depends on the presence of stimulatory glucose concentrations. Although this was an interesting observation that deserved further investigation, we focused our study on the mechanisms behind BPA regulation of Ca^2+^ entry at non-stimulatory glucose levels, in order to understand how the NMDR was generated.

### BPA decreased R-Type Ca^2+^ currents in a non-monotonic manner

**Figure 3:**
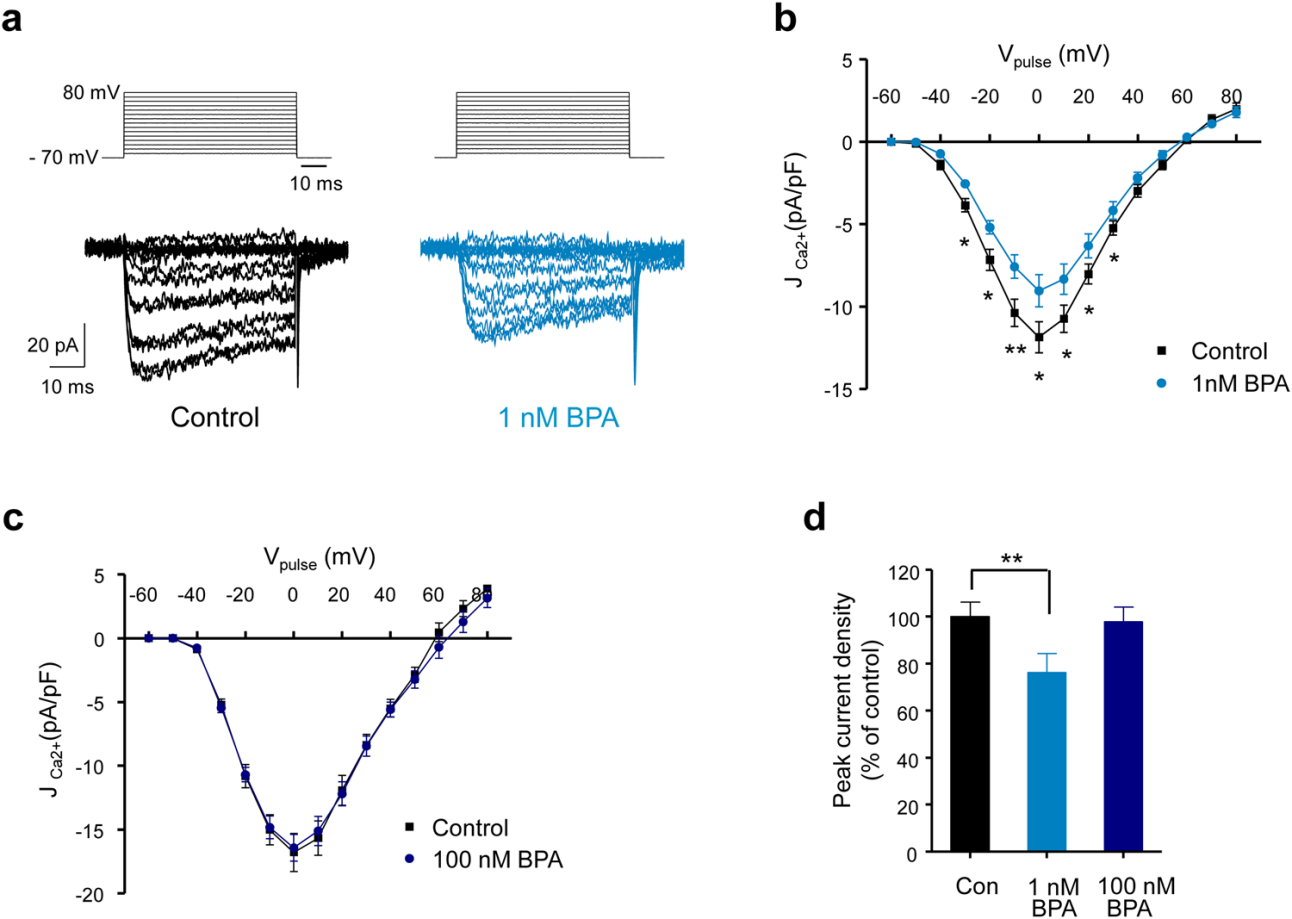
BPA inhibits Ca^2+^ currents in a non-monotonic manner in mouse pancreatic β-cells. (**a**) Representative recordings of Ca^2+^ currents (lower panels) in response to depolarizing voltage pulses (−60 mV to +80 mV from a holding potential of −70 mV, 50 ms duration; upper panels) in control (left panel; black traces) and 1 nM BPA-treated (right panel; blue traces) isolated β-cells. (**b**) Average relationship between Ca^2+^ current density (J, Ca^2+^ currents normalized to the cell size in pF) and the voltage of the pulses in the control (black squares, *n* = 33) and 1 nM BPA-treated (blue circles, *n* = 33) cells. (**c**) The same experiment as in (**a**,**b**) but performed in β-cells cultured in the presence of 100 nM BPA (dark blue circles) (*n* = 12 cells/group). (**d**) Average normalized values of current density evoked at 0 mV obtained from the *I*–*V* relationship shown in (**b**) and (**c**). Data are represented as the mean ± s.e.m. Student’s *t*-test: *P ≤ 0.05; **P < 0.01. In (**d**). Mann-Whitney test compared to control: **P < 0.01.

To study how BPA decreased Ca^2+^ entry, we recorded Ca^2+^ currents using the patch-clamp technique in a whole cell configuration. Figure 3a shows the recordings of the Ca^2+^ currents in response to depolarizing voltage pulses from −60 to +80 mV from a holding potential of −70 mV. The recordings from the cells treated with 1 nM BPA for 48 hours showed smaller currents than vehicle-treated cells (Fig. 3a). Currents were significantly smaller in the presence of BPA in response to depolarizing pulses from −30 to +30 mV (Fig. 3b), showing a maximum at approximately 0 mV, which was near the peak of the glucose-induced action potentials (see Fig. 1b). However, the Ca^2+^ currents were not modified in cells treated with 100 nM BPA (Fig. 3c). Therefore, as shown in Fig. 2d,e for the Ca^2+^ signals, BPA decreased the Ca^2+^ currents at a low dose of 1 nM but not at a high dose of 100 nM (Fig. 3d). This effect was similar when the natural hormone, 17β-oestradiol, was used instead of BPA (Supplementary Fig. 2).

**Figure 4:**
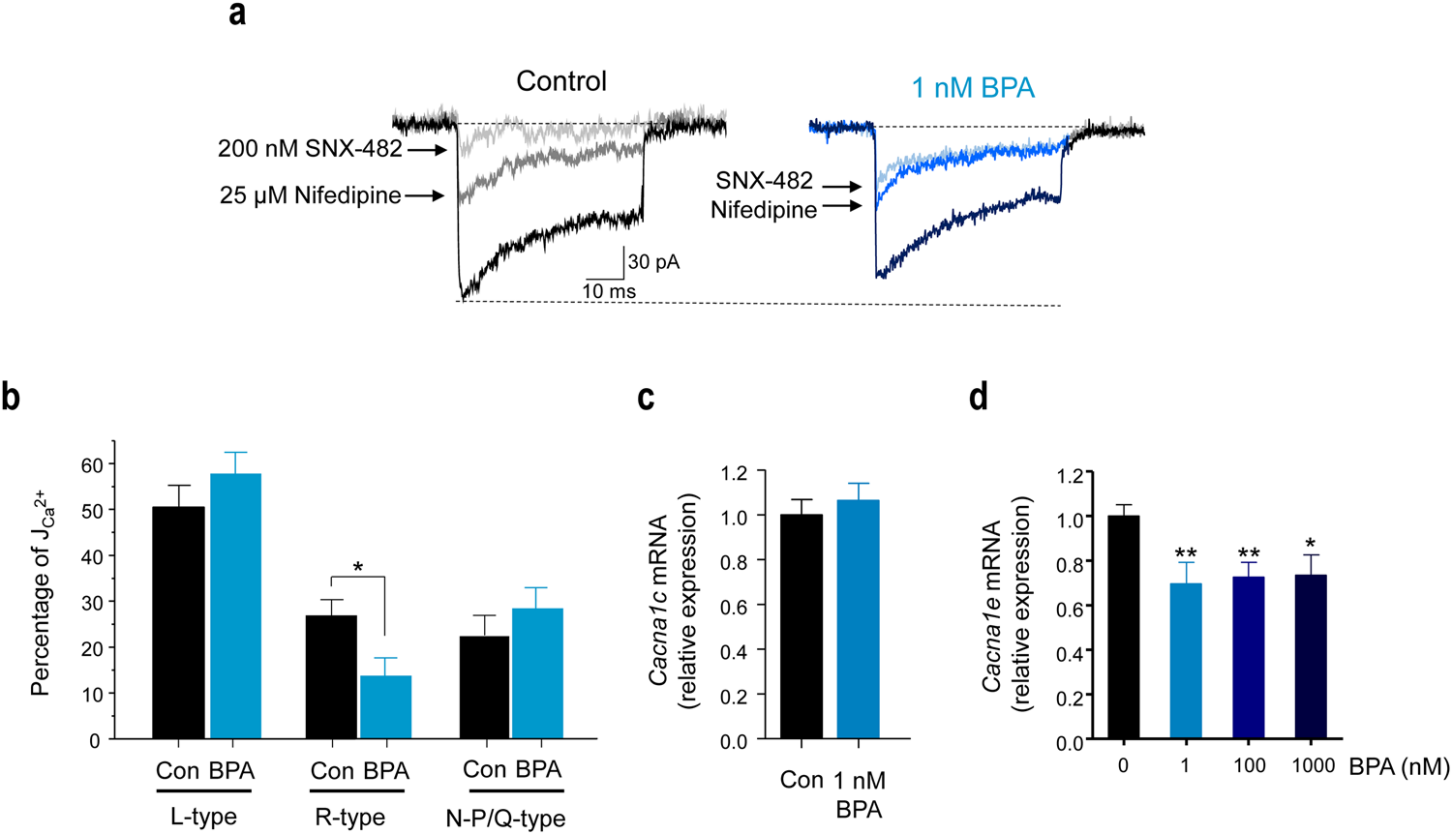
BPA inhibits R-type Ca^2+^ currents in mouse pancreatic β-cells. (**a**) Representative recordings of the Ca^2+^ currents in isolated β-cells in response to a depolarizing voltage pulse (−70 to 0 mV, 50-ms duration) in the absence of specific blockers (whole current), in the presence of 25 μM nifedipine (L-type current component blocker) and in the presence of 25 μM nifedipine +200 nM SNX-482 (R-type current component blocker) applied sequentially to the control (left panel) and 1 nM BPA-treated (right panel) cells. (**b**) Average Ca^2+^ current density percentage of the different components (compared to whole Ca^2+^ current density) in control (black bars, *n* = 8 cells) and in the presence of 1 nM BPA (blue bars, *n* = 9 cells). (**c**) Relative Ca_v_1.2 (*Cacna1c*) mRNA expression in the control (black bar) or 1 nM BPA-treated (blue bar) whole islets. Expression was normalized to the housekeeping gene Hprt1 (control, *n* = 8; 1 nM BPA, *n* = 7; obtained from 3 independent experiments). (**d**) Relative Ca_v_2.3 (*Cacna1e*) mRNA expression in islets exposed to 1 nM (*n* = 12), 100 nM (*n* = 13) and 1000 nM BPA (*n* = 7) for 48 hours, relative to control expression (*n* = 35; obtained from 12 experiments). Expression was normalized to the housekeeping gene Hprt1. Student’s *t*-test: *P ≤ 0.05; **P < 0.01.

To determine which Ca^2+^ current subtypes were affected by 1 nM BPA, we used specific blockers of L- and R-type Ca^2+^ currents and measured the resulting current in response to one 50-ms voltage pulse from −70 to 0 mV, repeated every 10 s. Because a run-down of the Ca^2+^ currents in the whole-cell configuration has been reported^31^, we waited for the stabilization of the size of the current before we applied the specific blockers. When we used the L-type Ca^2+^ current blocker nifedipine (25 μM), the whole current was reduced by 50%, and it was unmodified by the BPA treatment (Fig. 4a,b). In addition to nifedipine, we added the R-type Ca^2+^ current blocker SNX-482 (200 nM), which further reduced the remaining whole current by 25% in the control cells (Fig. 4a,b). Notably, in cells exposed to BPA, SNX-482 blocked only a small amount of current, indicating that the R-type current was already reduced (Fig. 4a,b), presumably because of BPA exposure. SNX-482 also abolished the BPA-induced reduction in the Ca^2+^ entry measured in isolated β-cells treated with high extracellular K^+^ (data not shown). The percentage of Ca^2+^ current flowing through the N-P/Q-type Ca^2+^ channels was unaltered by BPA (Fig. 4b). These results strongly indicated that BPA exposure specifically reduced the R-type Ca^2+^ current. This may occur via activation of a signalling pathway that modulates the biophysical characteristics of the channel and/or after transcriptional regulation of the channel subunits. Therefore, we measured BPA effects on the mRNA expression of Cav2.3 (*Cacna*1*e*), the pore forming α subunit responsible for the R-type Ca^2+^ channel, and the mRNA expression of Cav1.2 (*Cacna1c*), the pore forming α subunit responsible for the L-type Ca^2+^ channel. BPA at 1 nM significantly decreased Cav2.3 expression (Fig. 4d), but it had no effect on Cav1.2 (Fig. 4c). This experiment indicated that 48 hours of BPA treatment between 1 nM and 1 µM reduced Cav2.3 transcription to similar levels.

### ERβ is involved in BPA regulation of the R-Type Ca^2+^ current and Cav2.3 transcription

**Figure 5:**
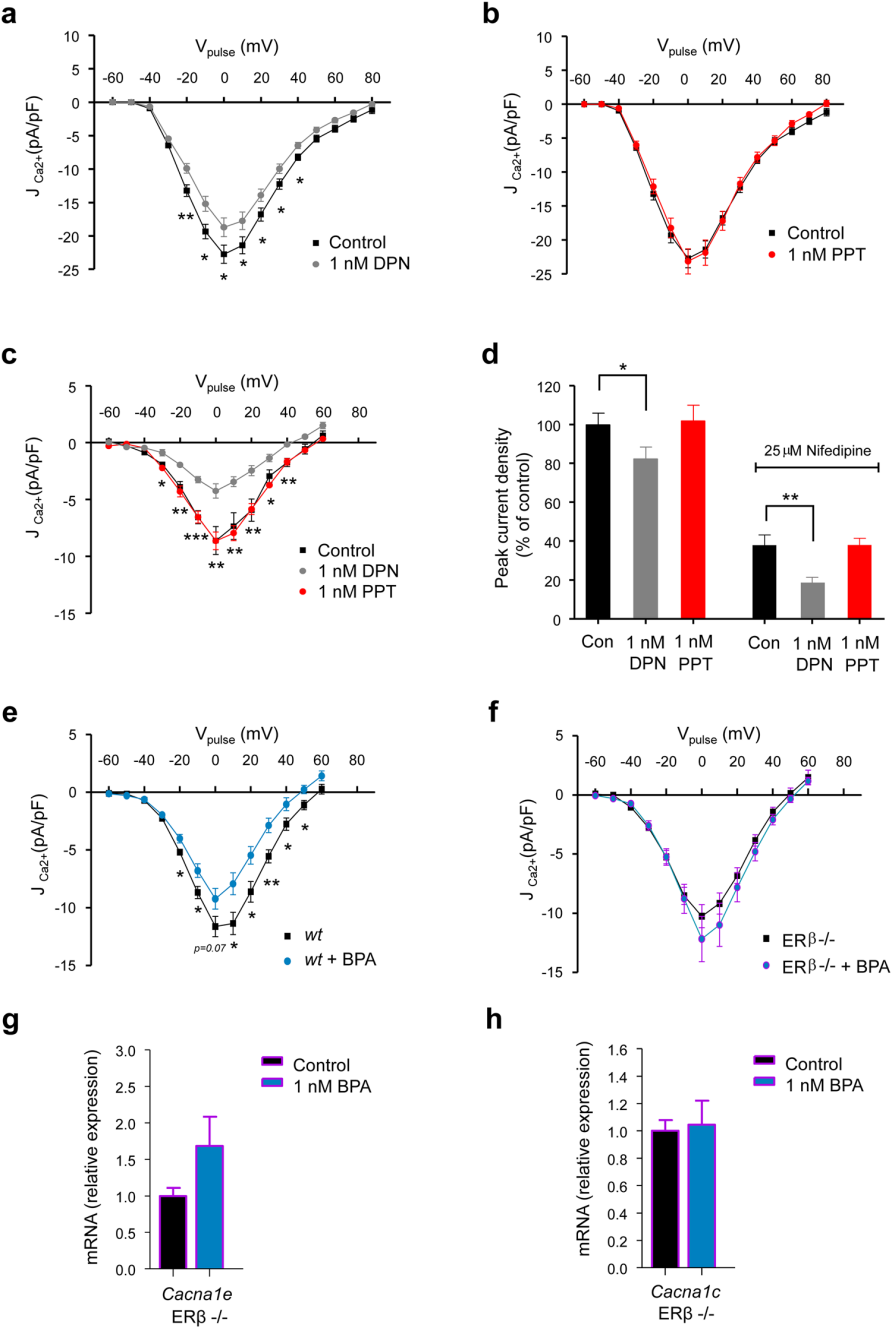
BPA-induced inhibition of R-Type Ca^2+^ current and Ca_v_2.3 transcription involves oestrogen receptor β. (**a**) Average relationship between the Ca^2+^ current density and the voltage of the pulses in β-cells (*Control*; black squares, *n* = 20) or treated with 1 nM DPN for 48 hours (grey circles, *n* = 21). (**b**) The same experiment as in (**a**) but using β-cells treated with 1 nM PPT (red symbols, *n* = 16) for 48 hours. (**c**) Average relationship between the Ca^2+^ current density and the voltage of the pulses in the presence of 25 μM nifedipine. Control β-cells (*Control*; black squares, *n* = 6) or cells treated with 1 nM DPN (grey circles, *n* = 7) or 1 nM PPT (red circles, *n* = 5) for 48 hours. (**d**) Histogram summarizing the average normalized peak Ca^2+^ current density shown in panels (a),(b) and (c). (**e**) Average relationship between the Ca^2+^ current density and the voltage of the pulses in the presence of 25 μM nifedipine. Pancreatic β-cells from wild-type mice were left untreated (*wt*; black squares, *n* = 12) or treated with 1 nM BPA (*wt* + *BPA*; blue circles, *n* = 18 cells) for 48 hours. (**f**) The same experiment as in (**e**) but with β-cells from ERβ^−/−^ mice (*ERβ*^−/−^; black squares, *n* = 11 cells; *ERβ*^−/−^ + *BPA*; blue circles, *n* = 11 cells). (**g**) Relative Ca_v_2.3 (*Cacna1e*) mRNA expression in whole islets from ERβ^−/−^ mice. Islets were untreated (black bar) or treated with 1 nM BPA (blue bar) for 48 hours. Expression was normalized to the housekeeping gene Hprt1. (**h**) As in (**g**) but showing the relative mRNA expression of Ca_v_1.2 (*Cacna1c* gen). In the measurements shown in **g**,**h**, the RNA samples were obtained from the ERβ^−/−^ islets of 3 independent experiments (*Control*; black bars, *n* = 6; *1* *nM BPA*; blue bars, *n* = 6). Data are represented as the mean ± s.e.m. Student’s *t*-test: *P ≤ 0.05; **P < 0.01; ***P < 0.001.

BPA acts in β-cells via ERβ and ERα^32^. To evaluate the possible roles of these receptors in the regulation of the R-type Ca^2+^ current described above, we incubated β-cells with agonists of ERβ (DPN) and ERα (PPT). DPN (1 nM) imitated 1 nM BPA in reducing the Ca^2+^ currents (Fig. 5a), while 1 nM PPT had no effect (Fig. 5b). This was better visualized when we removed the L-Type component of the Ca^2+^ current using nifedipine (Fig. 5c). These results are summarized in Fig. 5d, in which we represent the percentage of the current density in response to a voltage pulse from −70 mV to 0 mV. Note how the total Ca^2+^ current is reduced by approximately 20% by DPN at 1 nM (left bars), while the residual current in the presence of nifedipine decreased by approximately 50% (right bars). When we tested these agonists on the depolarization-induced [Ca^2+^]_i_ increase, we obtained similar results (Supplementary Fig. 3). The decrease in [Ca^2+^]_i_ induced by 1 nM BPA was abolished using the ERβ antagonist PHTPP but was unaffected by the ERα antagonist MPP (Supplementary Fig. 3). These experiments strongly suggest the involvement of ERβ in the response to low BPA concentrations (1 nM). To further demonstrate the implications of ERβ, we performed the same experiment as in Fig. 5c but used β-cells from wild-type and ERβ−/− mice. In the β-cells from the wild-type mice, BPA reduced the Ca^2+^ currents (Fig. 5e), but the BPA effect was absent in the cells from the ERβ−/− mice (Fig. 5f). When we measured the mRNA levels in the islets from the ERβ−/− mice, 1 nM BPA had no effect on *Cacna1e* expression. Regardless, it had a tendency to increase rather than decrease (Fig. 5g). The mRNA levels of *Cacna1c* remained unchanged (Fig. 5h). These experiments indicated that ERβ was involved in the regulation of *Cacna1e* transcription and likely involved, as a consequence, in decreasing the R-type Ca^2+^ currents.

### Activation of ERα opposes ERβ actions on Ca^2+^ currents

**Figure 6:**
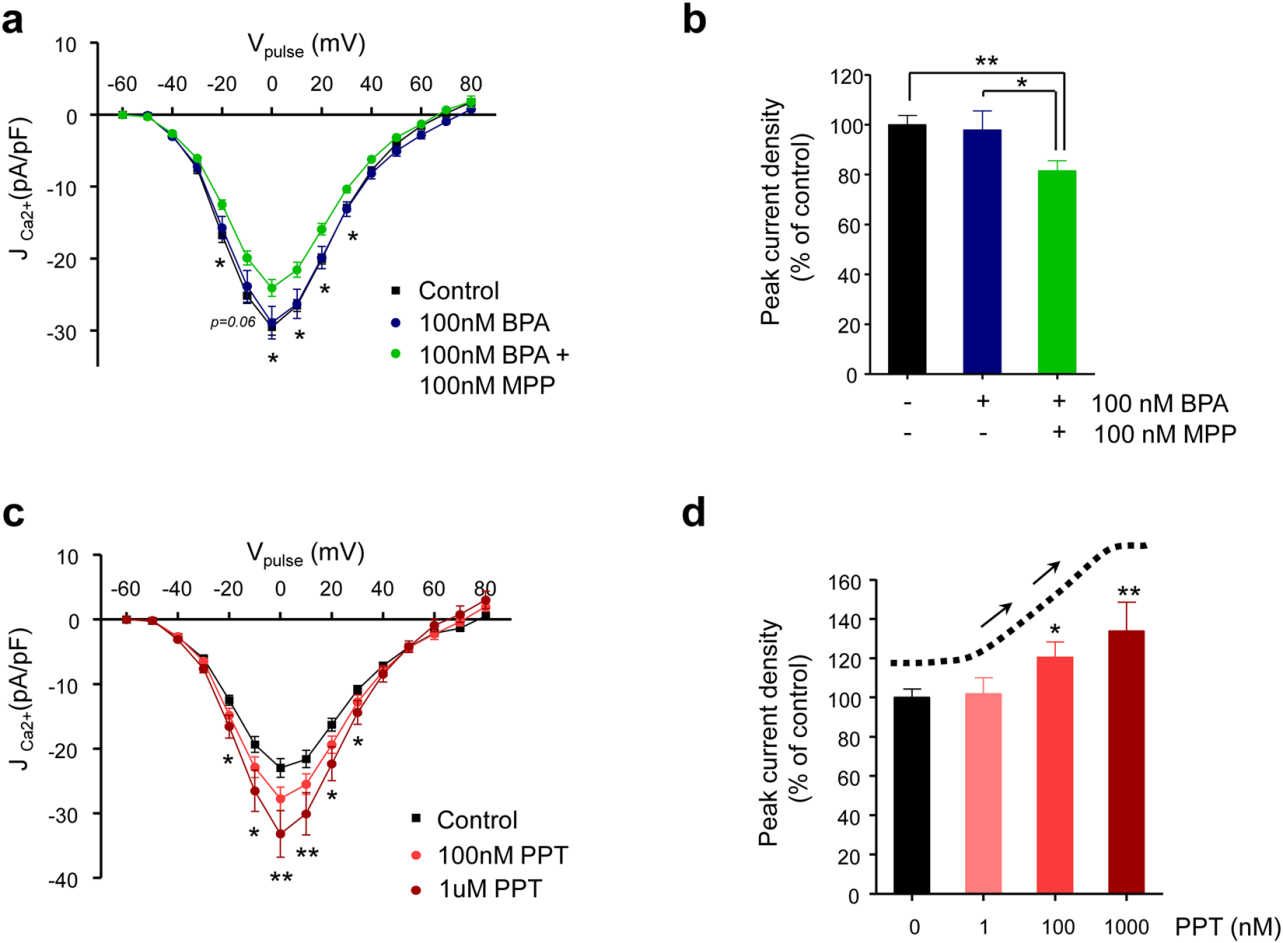
Activation of oestrogen receptors α and β produces opposing actions on the Ca^2+^ currents in mouse pancreatic β-cells. (**a**) Average relationship between the Ca^2+^ current density and the voltage of the pulses in β-cells left untreated (*Control*; black squares, *n* = 14) or treated with 100 nM BPA (100 *nM BPA*; dark blue circles, *n* = 9) or 100 nM BPA plus 100 nM MPP (100 *nM BPA* + 100 *nM MPP*; green symbols, *n* = 18) for 48 hours. (**b**) Histogram summarizing the average normalized peak Ca^2+^ current density shown in panel (a). (**c**) Average relationship between the Ca^2+^ current density and the voltage of the pulses in pancreatic β-cells (*Control*; black squares, *n* = 26) or treated with 100 nM (100 *nM PPT*; light red circles, *n* = 21) or 1000 nM PPT (100 *nM PPT*; dark red circles, *n* = 9) for 48 hours. (**d**) Histogram summarizing the average values of the peak Ca^2+^ current density in vehicle-treated β-cells (*Control*) or cells treated with 1 nM PPT (control, *n* = 20; 1 nM PPT, *n* = 16), 100 nM PPT (control, *n* = 26; 100 nM PPT, *n* = 21), or 1000 nM PPT (control, *n* = 14; 1000 nM PPT, *n* = 9), normalized to the peak density of the corresponding experimental control cells. The dotted line and arrows depict the monotonic dose-response relationship at the top of the histogram. Data are represented as the mean ± s.e.m. Student’s *t*-test: *P ≤ 0.05; **P < 0.01. (**a**) 100 nM BPA vs. 100 nM BPA + 100 nM MPP. (**c**) Control vs. 1000 nM PPT. (**d**) Paired comparison vs. control.

The divergence between the observed effect of BPA on Ca^2+^ entry, which showed NMDR behaviour (see Fig. 2d,e), and the MDR relationship between BPA and *Cacna1e* expression (see Fig. 4d) strongly suggests the existence of another mechanism that is activated at high doses to explain the non-monotonicity. Because ERα is involved in important signalling processes triggered by BPA in β-cells^19^, we performed the experiment depicted in Fig. 6a to test whether ERα is implicated in the regulation of Ca^2+^ entry. For this purpose, we incubated β-cells with the ERα antagonist MPP to block any possible ERα effect on the Ca^2+^ currents. When ERα was blocked, 100 nM BPA decreased the Ca^2+^ currents to a similar extent as 1 nM BPA, yet 100 nM BPA in the absence of MPP had no effect (Figs 3c,d and 6a,b). This result suggests that ERα may counteract the ERβ reduction of Ca^2+^ entry by increasing the Ca^2+^ currents in response to 100 nM BPA. Note that we did not use cells from the ERα−/− mice because these mice were obese and insulin-resistant from an early age^33^, and obesity changes ion channel activity, calcium signalling and exocytosis^34^. To further demonstrate the role of ERα on Ca^2+^ current potentiation we exposed cells to increasing doses of the ERα agonist PPT. Figure 6c,d, demonstrates that 100 nM and 1 µM PTT increased the Ca^2+^ currents. This occurred along a wide voltage range (Fig. 6c) in an MDR manner (Fig. 6d).

**Figure 7:**
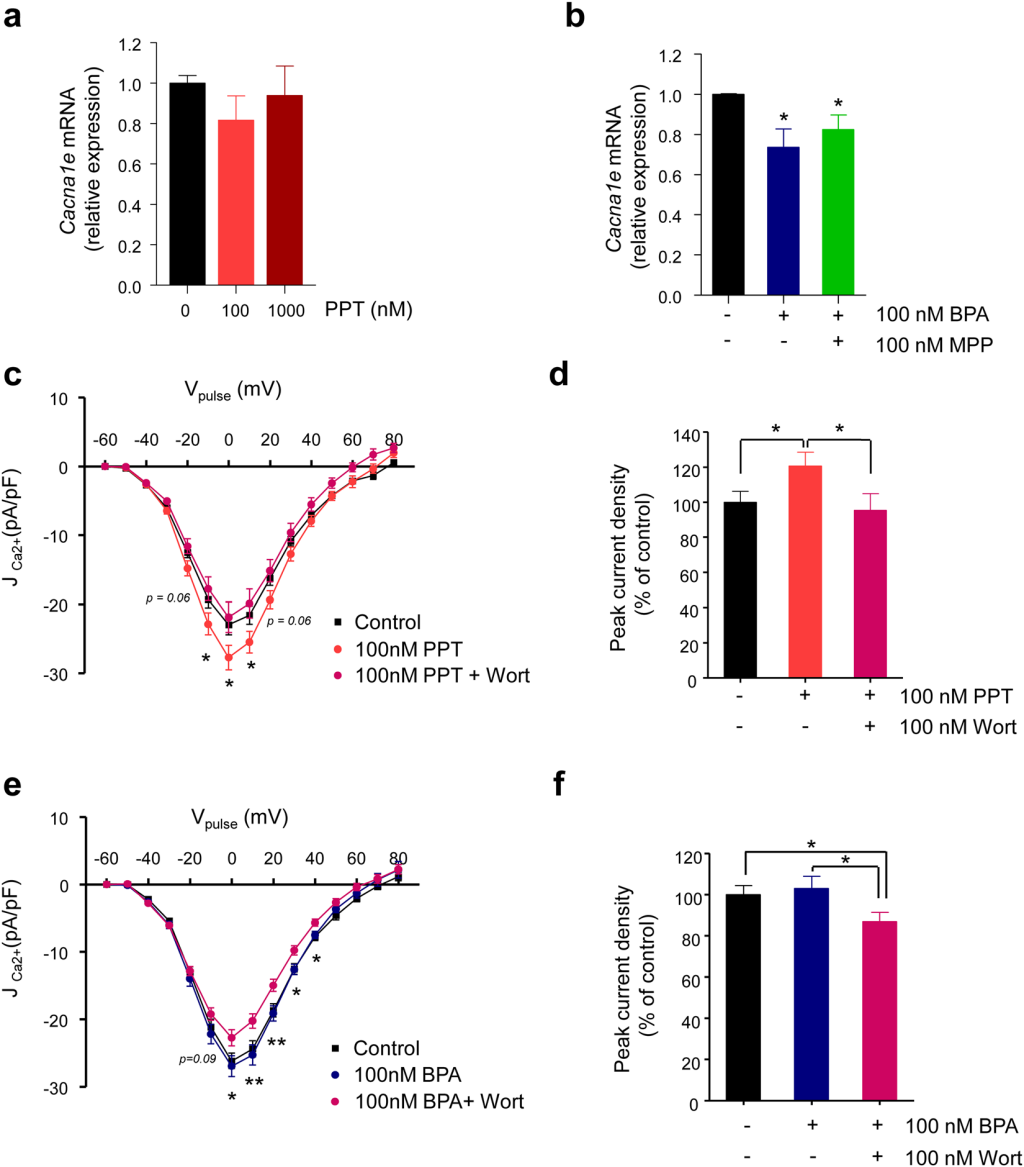
Potentiation of the Ca^2+^ currents by BPA through oestrogen receptor α activation involves PI3K. (**a**) Relative Ca_v_2.3 (*Cacna1e*) mRNA expression in control islets (black bar, *n* = 13) or islets exposed to 100 nM PPT (light red bar, *n* = 11) or 1000 nM PPT (dark red bar, *n* = 11). Expression was normalized to the housekeeping gene HPRT. (**b**) Relative Ca_v_2.3 (*Cacna1e*) mRNA expression in control islets (black bar, *n* = 7) or islets exposed to 100 nM BPA (dark blue bar, *n* = 7) or 100 nM BPA + 100 nM MPP (green bar, *n* = 7). (**c**) Average relationship between the Ca^2+^ current density and the voltage of the pulses in β-cells left untreated (*Control*; black squares, *n* = 26) or treated with 100 nM PPT (100 *nM PPT*; light red circles, *n* = 21) or 100 nM PPT + 100 nM wortmannin (100 *nM PPT* + *Wort*; magenta circles, *n* = 12). (**d**) Histogram summarizing the average normalized peak Ca^2+^ current density shown in panel (**c**). (**e**) Average relationship between the Ca^2+^ current density and the voltage of the pulses in β-cells left untreated (*Control*; black squares, *n* = 12) or treated with 100 nM BPA (100 *nM BPA*; dark blue circles, *n* = 15) or 100 nM BPA plus 100 nM wortmannin (100 *nM BPA* + *Wort*; magenta circles, *n* = 16). (**f**) Histogram summarizing the average normalized peak Ca^2+^ current density shown in panel (**e**). RNA samples used in (**a**) and (**b**) were obtained from the islets of 3–5 independent experiments. Data are represented as the mean ± s.e.m. Student’s *t*-test: *P ≤ 0.05; **P < 0.01. (**b**) Paired comparison with respect to control expression. (**c**) 100 nM PPT vs. 100 nM PPT + 100 nM wortmannin. (**e**) 100 nM BPA vs. 100 nM BPA + 100 nM wortmannin.

The potentiation of the Ca^2+^ currents by high doses of either PPT or BPA might be due to an effect that is opposite to that of ERβ on *Cacna1e* expression. To test this hypothesis, we measured *Cacan1e* mRNA in response to 100 nM and 1 µM PPT, but no changes were observed upon PPT exposure (Fig. 7a). Furthermore, the 100 nM BPA-induced decrease in the mRNA *Cacan1e* levels remained unchanged in the presence of the ERα blocker MPP (Fig. 7b). Although we cannot rule out transcriptional downregulation of other Ca^2+^ channel genes, this result points to an effect of ERα activation beyond *Cacan1e* expression. Therefore, ERα does not counteract the transcriptional effect of ERβ at high BPA concentrations.

In addition to the role of ERα in the regulation of gene expression, it is well known that ERα acts outside of the cell nucleus by activating PI3K and ERK1/2 signalling cascades, among others^35–37^. In β-cells, for instance, ERα activation of ERK1/2 regulates insulin biosynthesis^19,38^. To investigate whether these kinases play a role in the potentiation of Ca^2+^ entry, we measured the Ca^2+^ currents in cells incubated with PPT at 100 nM to activate ERα, in the presence and absence of the broad-spectrum PI3K inhibitor wortmannin (Fig. 7c–f and Supplementary Fig. 4). We observed that the 100 nM PPT-induced potentiation of the Ca^2+^ currents was abolished by wortmannin, while wortmannin itself had no effect on the Ca^2+^ currents (Fig. 7c–f and Supplementary Fig. 4).

To demonstrate that PI3K was involved in the response to 100 nM BPA, we performed the experiment shown in Fig. 7e. Cells incubated with the vehicle (control) or 100 nM BPA showed identical Ca^2+^ currents. However, cells incubated in the presence of 100 nM BPA plus wortmannin showed a decreased Ca^2+^ current. The ERK1/2 blocker PD98059 had no effect on the potentiation of the Ca^2+^ currents by 100 nM PPT (Supplementary Fig. 4). This experiment indicates that the potentiation of the Ca^2+^ currents by high doses of BPA may involve PI3K.

## Discussion

Non-monotonic dose response curves have been demonstrated for natural hormones and EDCs in a variety of biological systems^39,40^. However, a proper understanding of NMDRs for EDCs, including the underlying molecular mechanisms, remains elusive. The findings obtained here indicate that the highly produced and ubiquitous EDC, BPA, alters the shape of glucose-induced action potentials in β-cells by decreasing its amplitude and increasing their width. The amplitude of the action potentials in mouse β-cells depends on voltage-gated Ca^2+^ channels. Therefore, a decreased amplitude should be correlated with diminished Ca^2+^ entry. This was manifested when Ca^2+^ signals in response to glucose and extracellular K^+^ depolarization were measured in whole islets. Remarkably, the relationship between [Ca^2+^] signaling, elicited by a high extracellular K^+^ depolarization and BPA, was non-monotonic, i.e., Ca^2+^ signaling decreased in response to low BPA concentrations (100 pM and 1 nM), but higher concentrations (10 and 100 nM) produced no effect (Fig. 2d,e). We should note that 1 nM BPA is approximately the concentration of BPA in serum of USA population^9,17^, while higher levels between 10 and 100 nM may be expected in industry workers using BPA^18^.

As described in the introduction, this type of NMDR curve is commonly obtained in response to EDC exposure, and this NMDR curve type has tremendous implications in the whole field of toxicology and particularly, in regulatory toxicology^17,40,41^. We decided, therefore, to focus on understanding the molecular mechanisms underlying this behaviour.

The decrease in Ca^2+^ signalling provoked by a low dose of BPA (1 nM) was associated with a reduction in the Ca^2+^ currents in response to depolarizing voltage pulses (Fig. 3a,b). The pharmacological dissection indicated that the channel that was downregulated by 1 nM BPA was Cav2.3, which is responsible of the R-type Ca^2+^ current and represents 25% of the whole Ca^2+^ current^42^ (Fig. 4a,b). The decreased of R-type Ca^2+^ currents after BPA exposure were likely a consequence of transcriptional downregulation of the *Cacna1e* gene, which encodes the alpha-1E subunit of the Cav2.3 channels (Fig. 4b). The consequences of downregulating *Cacna1e* expression were similar, although not identical, to those described in β-cells from the Cav2.3−/− mice^29^. In both the Cav2.3−/− mice and our BPA model, there was a decrease in the glucose-induced Ca^2+^ response in the whole islets of Langerhans. However, at the single β-cell level, the capacitance experiments performed here indicated that exocytosis was diminished from the initial component. This is slightly different from the situation found in the Cav2.3−/− mice, in which the later component of exocytosis was decreased while the first component was not affected much^29^. Therefore, we cannot discard other effects that are unrelated to downregulation of Cav2.3 gene expression in the modulation of the exocytotic pathways. Interestingly, we found that the consequences of the BPA treatment on insulin exocytosis and secretion were different in the absence and presence of glucose. In the absence of glucose, there was decreased Ca^2+^ entry in response to extracellular K^+^-induced membrane depolarization. This was likely responsible for the diminished exocytosis and the decreased insulin release in response to high extracellular K^+^ in BPA-treated cells. In contrast, insulin release and exocytosis in the presence of 11 mM glucose were potentiated by a 1-nM BPA incubation, despite the decreased glucose-induced calcium signals (Supplementary Fig. 1c–e). This highly suggests that BPA also regulated the exocytotic machinery independently of Ca^2+^ entry. Glucose acts by increasing the concentration of Ca^2+^ and cAMP in the β-cell cytoplasm. Apart from the triggering role of Ca^2+^, insulin release is markedly amplified by cAMP, a second messenger required for normal insulin release^43^. In cardiac myocytes 1 nM BPA increases cAMP production^44^, therefore it is possible that glucose-induced increase in cAMP is amplified in β-cells after incubation with 1 nM BPA. Further research is necessary to mechanistically understand this effect.

The decrease in both the R-type Ca^2+^ currents and *Cacna1e* gene expression by 1 nM BPA implicated ERβ (Fig. 5). This is a BPA action triggered at low concentrations, similar to that found in human serum^45^ and far below concentrations used in classical toxicology^9^. For many years, it has been assumed that BPA behaved as a weak oestrogen. This was, in part, because *in vitro* experiments indicated that ERβ binds BPA with much lower affinity than the natural hormone, E2. The dissociation constant (K_d_) for BPA upon binding to ERβ was approximately 10–40 µM, and its binding induced transcriptional activity with a potency between 1000 and 5000-fold lower than E2, starting within the micromolar range^46,47^. These results, however, could not explain the *in vivo* data that indicated that BPA acted at concentrations within the nanomolar range^48^. During the past two decades, numerous studies have accumulated data describing the actions of BPA *in vitro* at concentrations of approximately 1 nM^10,19,20,49^. The view of BPA as a weak oestrogen was based on the classical model in which either ERα or ERβ regulated gene transcription after binding to an oestrogen response element (ERE) in DNA and recruiting co-regulators^50^. Currently, our knowledge of oestrogen signalling has advanced, and it is accepted that both oestrogen receptors act through non-ERE-dependent pathways and activate signalling cascades outside of the nucleus^37^. It is through these pathways that BPA behaves as a potent oestrogen and triggers effects within the picomolar and nanomolar range^32,51^. Because the *Cacna1e* gene does not have an ERE sequence (result obtained using Transfac^52^), its transcription must be downregulated by a non-ERE-dependent pathway. This can explain why BPA triggers transcriptional downregulation of *Cacna1e* at low dose such as 1 nM.

It is noteworthy that *Cacan1e* expression decreased within a whole range of BPA concentrations, from 1 nM to 1 µM, but the Ca^2+^ currents were diminished only with 1 nM BPA. Therefore, another mechanism must account for the absence of the effect at 100 nM BPA. Because the Ca^2+^ currents were potentiated in ERβ−/− cells in the presence of BPA, we pursued the involvement of another oestrogen receptor in the non-monotonic response to BPA. Mouse pancreatic β-cells express ERα, ERβ and G protein-coupled oestrogen receptor 1 (GPER), and these receptors play roles in the regulation of insulin content, insulin secretion and β-cell survival^53–58^.

The present data suggest BPA doses of 100 nM and 1 µM potentiate Ca^2+^ currents by an ERα-dependent mechanism (Fig. 6). Evidence has indicated that ERα acts outside of the nucleus to activate PI3K to directly or indirectly enhance Ca^2+^ currents. ERα activates ERKs and PI3K in many cellular systems^59,60^ including β-cells^19,38^. PI3K also regulates Ca^2+^ channels in other excitable cells and promotes calcium channel trafficking to the plasma membrane^61^. Which Ca^2+^ channel types are regulated here by BPA is a subject for further research, but the whole Ca^2+^ current is enlarged in response to 100 nM BPA via ERα- and PI3K-mediated counteractions of the downregulation produced by ERβ (Fig. 7).

**Figure 8:**
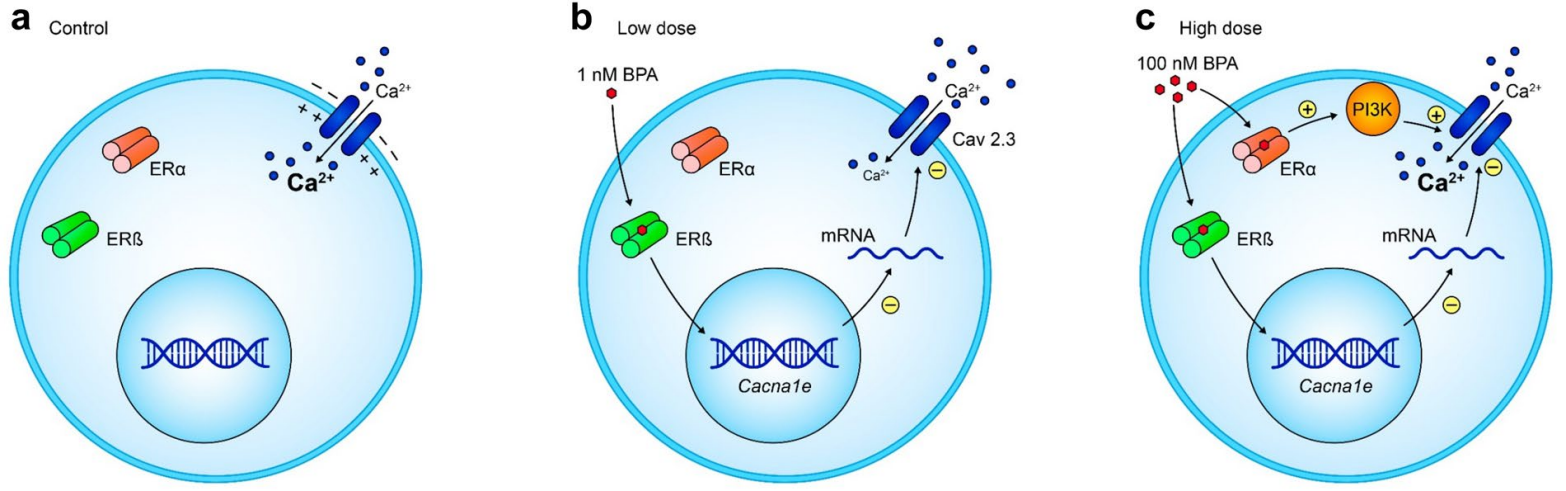
Cellular Model of the NMDR relationship between BPA and Ca^2+^ entry. (**a**) Depolarization of the plasma membrane opens voltage-gated Ca2+ channles and induces Ca2+ influx (**b**) Low concentrations of BPA (100 pM-1 nM) decrease *Cacna1e* expression and Ca^2+^ entry in response to plasma membrane depolarization. (**c**) Higher BPA concentrations (100 nM-1 µM) activate ERα, which in turn enhances the Ca^2+^ currents in a PI3K-dependent manner. As the actions of ERβ on *Cacna1e* transcription remain during BPA exposure, the outcome is an unchanged Ca^2+^ entry.

In summary, the results presented in this study provide a mechanistic explanation for the NMDR relationship between BPA exposure and Ca^2+^ entry. A model is proposed in Fig. 8. Low concentrations of BPA between 0.1–1 nM act via ERβ to transcriptionally downregulate Cav2.3 gene expression and are responsible for the decrease in the whole Ca^2+^ current measured in patch-clamp experiments and for the high extracellular K^+^-induced Ca^2+^ entry recorded in the Fura-2 experiments (Fig. 8b). The dynamic with higher BPA concentrations (100 nM-1 µM) involves both ERβ and ERα. ERβ at 100 nM BPA downregulates Cav2.3 gene expression and decreases the whole Ca^2+^ current, but ERα simultaneously potentiates whole Ca^2+^ currents through a PI3K-dependent pathway (Fig. 8c). Both effects counteract each other, and when Ca^2+^ currents or [Ca^2+^]_i_ are measured as end points, there is no net effect of the high BPA concentrations. Therefore, we have demonstrated that this NMDR curve is the outcome of at least two different mechanisms activated by low and high doses of BPA involving ERβ and ERα.

It is important to note that NMDR can arise from different molecular mechanisms that are still greatly uncharacterized, but they are beginning to be uncovered. In the case of female cardiac myocytes, acute exposure to BPA (100 pM-1 µM) produced Ca^2+^ transients in an NMDR^62,63^, which was explained by a mechanism involving ERβ acting on two different end points, a potentiation of Ca^2+^ release and uptake from the sarcoplasmic reticulum and an inhibition of plasma membrane L-Type Ca^2+^ channels^63^.

The unveiling of NMDR molecular mechanisms should have a high impact in the field of toxicology because the NMDR mechanisms demonstrate that dose and effect do not move together in a predictable manner. It cannot be always assumed that EDCs which are toxic at high doses are much less risky at lower, environmentally relevant levels. As a consequence, common concepts in regulatory toxicology such as the potency and threshold of effects cannot be easily applied in the case of BPA and, likely, other EDCs. Understanding the different mechanisms behind the NMDR should enable the development of new testing protocols to improve hazard and risk assessments.

## Methods

### Materials

Bisphenol-A was obtained from MP Biomedicals (Cat No 155118; Santa Ana, CA, USA). 17β-oestradiol (Cat No E8875), diazoxide (Cat No D9530), and nifedipine (Cat No D7634) were obtained from Sigma-Aldrich (Saint Louis, MO, USA). SNX-482 (Cat No 4363) was obtained from PeptaNova (Sandhausen, Germany). DPN (Cat No 1494), PPT (Cat No 1426), PHTPP (Cat No 2662), MPP dihydrochloride (Cat No 1991), PD98059 (Cat No 1213), and Wortmannin (Cat No 1232) were obtained from Tocris Cookson (Bristol, UK).

### Experimental animals and protocols

Adult C57BL/6 J male mice (10–14 weeks old) were used. All animals were kept under standard housing conditions. ERβ−/− mice were generated as described previously^64^ and supplied by Jan-Ake Gustafsson’s laboratory. All genetically modified and wild-type animals were obtained from the same supplier and the same colony. All experimental procedures were performed according to the Spanish Royal Decree 1201/2005 and the European Community Council directive 2010/63/EU. The Ethics Committee from Universidad Miguel Hernández de Elche (Alicante, Spain) approved all methods used in this study (approvals ID: UMH-IB-AN-01–14 and UMH-IB-AN-02-14). Animals were treated humanely and with care to alleviate suffering. All procedures were carried out in accordance with the approved guidelines and regulations.

### Culturing of islets and isolated islet cells

Pancreatic islets of Langerhans were isolated using collagenase (Sigma-Aldrich) as previously described^65^ and used according to the kind of experiment to be performed. The solution used for the isolation of the islets of Langerhans contained 115 mM NaCl, 5 mM KCl, 10 mM NaHCO_3_, 1.1 mM MgCl_2_, 1.2 mM NaH_2_CO_4_, 2.5 mM CaCl_2_, 25 mM HEPES, 5 mM Glucose, and 0.25% BSA (pH 7.4). When required, pancreatic islets were dispersed into isolated cells and cultured in RPMI 1640 (Gibco, Thermo Fisher Scientific) containing 11 mM glucose and without phenol red at 37 °C in a humidified atmosphere of 95% O_2_ and 5% CO_2_ for 48 hours. The medium was supplemented with 8% charcoal dextran-treated foetal bovine serum (HyClone, GE Health Care Life Sciences), 200 U/ml penicillin, 0.2 mg/ml streptomycin and 2 mM L-glutamine.

### Fluorometric Ca^2+^ measurements

Intracellular Ca^2+^ measurements were performed in whole islets or single islet cells loaded with 5 μM Fura-2AM (Molecular Probes, Eugene, OR, USA) for at least 1 hour at room temperature or 30 min at 37 °C, respectively, in a humidified atmosphere of 95% O_2_ and 5% CO_2_. Recordings were made using a constant-volume chamber with a controlled solution exchange and conducted at 35–37 °C. The regular bath solution contained 120 mM NaCl, 5 mM KCl, 25 mM NaHCO_3_, 1.1 mM MgCl_2_, 2.5 mM CaCl_2_ and 3 mM Glucose, pH 7.35 and was gassed with 95% O_2_ and 5% CO_2_. High extracellular K^+^ solutions, containing 20 mM or 65 mM KCl and 100 μM diazoxide, were used to elicit glucose-independent cell depolarization. Fluorescence measurements were obtained with an Axiovert 200 (Zeiss, Jana, Germany) inverted microscope. Images were acquired every 3 s with an extended high-resolution ORCA C4742-95 camera (Hamamatsu Photonics, Hamamatsu, Japan) using a dual-filter wheel (Sutter Instruments, Novato, CA, USA) equipped with 340 and 380 nm, 10-nm bandpass filters (Omega Optical, Brattleboro, VT, USA). Data acquisition was performed with the Aquacosmos 2.6 software (Hamamatsu Photonics). Cytosolic Ca^2+^ changes are represented as the ratio of the fluorescence emission intensities at 360 and 380 nm (F_360_/F_380_; fluorescence arbitrary units). The results were plotted and analysed using commercially available software (Origin, OriginLab Corporation, Northampton, MA, USA).

### Electrophysiology

A whole-cell patch-clamp configuration was used, except for the data in Fig. 1 in which the perforated patch whole-cell recording mode was applied. Pancreatic β-cells were identified by size (>5 pF) and the corresponding steady-state inactivation properties of the tetrodotoxin (TTX)-sensitive Na^+^ current^66^. Data were obtained using an Axopatch 200B patch-clamp amplifier (Axon Instruments Co. CA, USA). Patch pipettes were pulled from borosilicate capillaries (Sutter Instruments Co. CA, USA) using a flaming/brown micropipette puller P-97 (Sutter Instruments Co. CA, USA) and heat polished at the tip using an MF-830 microforge (Narishige, Japan). The bath solution for the voltage-gated Ca^2+^ current recordings contained 118 mM NaCl, 20 mM TEA-Cl, 5.6 mM KCl, 2.6 mM CaCl_2_, 1.2 mM MgCl_2_, 5 mM HEPES and 5 mM glucose (pH 7.4 with NaOH). The pipette solution for the voltage-gated Ca^2+^ current recordings consisted of 130 mM CsCl, 1 mM CaCl_2_, 1 mM MgCl_2_, 10 mM EGTA, 3 mM MgATP and 10 mM HEPES (pH 7.2 with CsOH). After filling the pipette with the pipette solution, the pipette resistance was 3–5 MΩ. A tight seal (>1 GΩ) was established between the β-cell membrane and the tip of the pipette by gentle suction. The series resistance of the pipette usually increased to 6–15 MΩ after moving to whole-cell. Series resistance compensation was used (up to 70%) for keeping the voltage error below 5 mV during current flow. In the recordings of the electrical activity in Fig. 1, the bath solution contained 140 mM NaCl, 3.6 mM KCl, 1.5 mM CaCl_2_, 0.5 mM MgCl_2_, 10 mM HEPES and 0–11 mM glucose (pH 7.4 with NaOH). The pipette solution for the electrical activity recordings consisted of (in mM) 76 K_2_SO_4_, 10 KCl, 10 NaCl, 1 MgCl_2_ and 5 HEPES (pH 7.35 with KOH). Amphotericin B (120–240 μg/ml; Sigma) was added to the pipette solution to perforate the patch^67^. The series resistance of the pipette usually increased to 20–30 MΩ after amphotericin-B perforation. Potential values were not corrected for the liquid junction potential. Voltage-gated Ca^2+^ currents were compensated for capacitive transients and linear leak using a -P/4 protocol. The experiments designed to measure exocytosis were performed on isolated β-cells using the standard whole-cell configuration. Exocytosis was detected as the change in cell capacitance, which was measured by the ‘sine + DC’ method^68^. The Jclamp software connected to an Axopatch 200B amplifier was employed in these experiments using the single-sine feature. The amplitude of the sine wave was 20 mV, and the frequency was set to 1.5 kHz. Sinus segments were interrupted to apply 10 square voltage pulses (from −70 to 0 mV with 500-ms durations) at 1 Hz to activate the voltage-gated Ca^2+^ entry that triggers exocytosis. In these experiments the bath solution was identical to the one used for the voltage-gated Ca^2+^ currents (see above) except that CaCl_2_ was increased to 5 mM. The pipette solution for the exocytosis measurements consisted of 140 mM CsCl, 10 mM NaCl, 1 mM MgCl_2_, 0.05 mM EGTA, 3 mM MgATP, 0.1 mM cAMP and 5 mM HEPES (pH 7.2 with CsOH). Pipettes in exocytosis experiments were coated with Sylgard 184 (Dow Corning Europe, Belgium) rubber to decrease the pipette capacitive transients. Data were filtered (2 kHz) and digitized (10 kHz) using a Digidata 1322 A (Axon Instruments Co. CA, USA) and stored in a computer for subsequent analysis using commercial software (pClamp9, Axon Instruments Co. CA, USA). All experiments were carried out at 32–34 °C.

### Insulin secretion and content

For the insulin secretion measurements, the 48 hours-cultured islets in the presence of vehicle or BPA (1 nM) were washed and kept in an incubator at 37 °C for 2 hours with a buffer solution containing 140 mM NaCl, 4.5 mM KCl, 2.5 mM CaCl_2_, 1 mM MgCl_2_, 20 mM HEPES, 3 mM D-glucose with a final pH of 7.4. Groups of 5 islets were then incubated in 400 μl of this buffer in the presence of 3, 8, or 16 mM glucose or 65 mM K^+^ in 3 mM glucose for 1 hour in the incubator at 37 °C. Afterwards, 100 μl of the buffer solution with the corresponding glucose concentration and 5% BSA was added, incubated at room temperature for 3 min and left to cool for 15 min on ice. Then, the medium was collected, and insulin was measured in duplicate samples using the Mouse Insulin ELISA Kit (Mercodia, Sweden). For the insulin content measurement, the islets grouped in batches of 5 were handpicked with a micropipette and incubated overnight in an ethanol/HCl buffer (75% Ethanol (v/v); 0.4% HCl (stock 37%) (v/v) and 24.6% distilled water (v/v)) at 4 °C. At the end of the incubation period, the buffer was removed and examined for insulin content using the Mouse Insulin ELISA Kit (Mercodia, Sweden)). Protein determination was performed using the Bradford dye method^69^. The insulin and protein contents were determined in each islet sample, and the ratio of both parameters was calculated for each sample.

### Real-time PCR

Pancreatic islets were isolated by collagenase digestion as described above and incubated in groups in RPMI 1640 in the presence of the stimuli for 48 hours. Quantitative PCR assays were performed using the CFX96 Real Time System (Bio-Rad, Hercules, CA, USA). Groups of 200–250 isolated islets were used for RNA extraction. RNA was extracted using a commercial kit (RNeasy Micro kit, Qiagen) according to the manufacturer’s instructions. RNA (0.5 μg) was reverse-transcribed using the High Capacity cDNA Reverse Transcription kit (Applied Biosystems, Foster City, CA, USA). Amplification reactions were carried out in medium containing 200 nM of each primer, 1 μl cDNA, and 1x IQ SYBR Green Supermix (Bio-Rad). HPRT was used as the housekeeping gene for the semiquantitative RT-PCR. PCR primers were as follows: *Cacna1e* forward, 5′-CCGATGATGATGAGAGGGAT-3′; *Cacna1e* reverse, 5′-TGCTGACTGTCTTCCAATGC-3′; *Cacna1c* forward, *5′*-GAGCCACGGTGAATCAGGA-3′; *Cacna1c* reverse, *5′*-GCAGTACTCGGCTTCTTCACTCA-3′; *Hprt1* forward, *5′*-GGTTAAGCAGTACAGCCCCA-3′; *Hprt1* reverse, *5′*-TCCAACACTTCGAGAGGTCC-3′. Samples were subjected to the following conditions: 3 minutes at 95 °C, 40 cycles (5 seconds at 95 °C, 5 seconds at 60 °C, and 10 seconds at 72 °C), and a melting curve of 65–95 °C. The resulting values were analysed with CFX Manager Version 1.6 (Bio-Rad) and were expressed as the relative expression with respect to the control values (2^−ΔΔCT^)^70^.

### Statistical analysis

The GraphPad Prism 5.0 software (GraphPad Software, Inc., CA, USA) was used for all statistical analyses. Data are expressed as the mean ± SEM. To assess differences between exposure groups, we used Student’s t-test or one-way analysis of variance (ANOVA) when appropriate. When data did not pass the parametric test, we used the Mann-Whitney and Kruskal-Wallis ANOVA tests (followed by Dunn’s test), depending on the experimental groups involved in the comparison. A probability level of ≤0.05 was considered statistically significant.

## Electronic supplementary material

Supplementary Figures
