# Author Correction: Molecular mechanisms involved in the non-monotonic effect of bisphenol-a on Ca^2+^ entry in mouse pancreatic β-cells

**DOI:** 10.1038/s41598-018-21309-w

**Published:** 2018-03-06

**Authors:** Sabrina Villar-Pazos, Juan Martinez-Pinna, Manuel Castellano-Muñoz, Paloma Alonso-Magdalena, Laura Marroqui, Ivan Quesada, Jan-Ake Gustafsson, Angel Nadal

**Affiliations:** 1 CIBER de Diabetes y Enfermedades Metabólicas Asociadas (CIBERDEM) and Institute of Bioenginering, Miguel Hernández University of Elche, Elche, Alicante Spain; 2 Departamento de Fisiología, Genética y Microbiología, Universidad de Alicante, Alicante, Spain; 3 Department of Cell Biology and Biochemistry, Center for Nuclear Receptors and Cell Signaling, University of Houston, Houston, Texas USA; 4 Department of Biosciences and Nutrition, Karolinska Institut, Huddinge, Sweden

Correction to: *Scientific Reports* 10.1038/s41598-017-11995-3, published online 18 September 2017

The original version of this Article contained an error in the title of the paper, where “Ca^2+^” was incorrectly given as “ca2+”. This has now been corrected in the PDF and HTML versions of the Article.

