# Supplementary material for: Molecular mechanisms involved in the non-monotonic effect of bisphenol-a on Ca2+ entry in mouse pancreatic β-cells
Source: Sci Rep. 2017 Sep 18;7:11770. doi: 10.1038/s41598-017-11995-3 (PMC5603522; doi:10.1038/s41598-017-11995-3)
Supplement: Supplementary file 1 — Supplementary Figures [file 41598_2017_11995_MOESM1_ESM.pdf]

SREP-17-30011-T

MOLECULAR MECHANISMS INVOLVED IN THE NON-MONOTONIC EFFECT OF BISPHENOL-A ON Ca2+ ENTRY IN MOUSE PANCREATIC β-CELLS

Sabrina Villar-Pazos*[1], Juan Martinez-Pinna*[2], Manuel Castellano-Muñoz[1], Paloma Alonso-Magdalena[1], Laura Marroquí[1], Ivan Quesada[1], Jan-Ake Gustafsson[3,4] and Angel Nadal[1]

[1]CIBER de Diabetes y Enfermedades Metabólicas Asociadas (CIBERDEM) and Institute of Bioenginering, Miguel Hernández University of Elche, Elche, Alicante, Spain. [2]Departamento de Fisiología, Genética y Microbiología, Universidad de Alicante, Alicante, Spain. [3]Department of Cell Biology and Biochemistry, Center for Nuclear Receptors and Cell Signaling, University of Houston, Houston, Texas, USA. [4]Department of Biosciences and Nutrition, Karolinska Institut, Huddinge, Sweden.

*Equal contribution

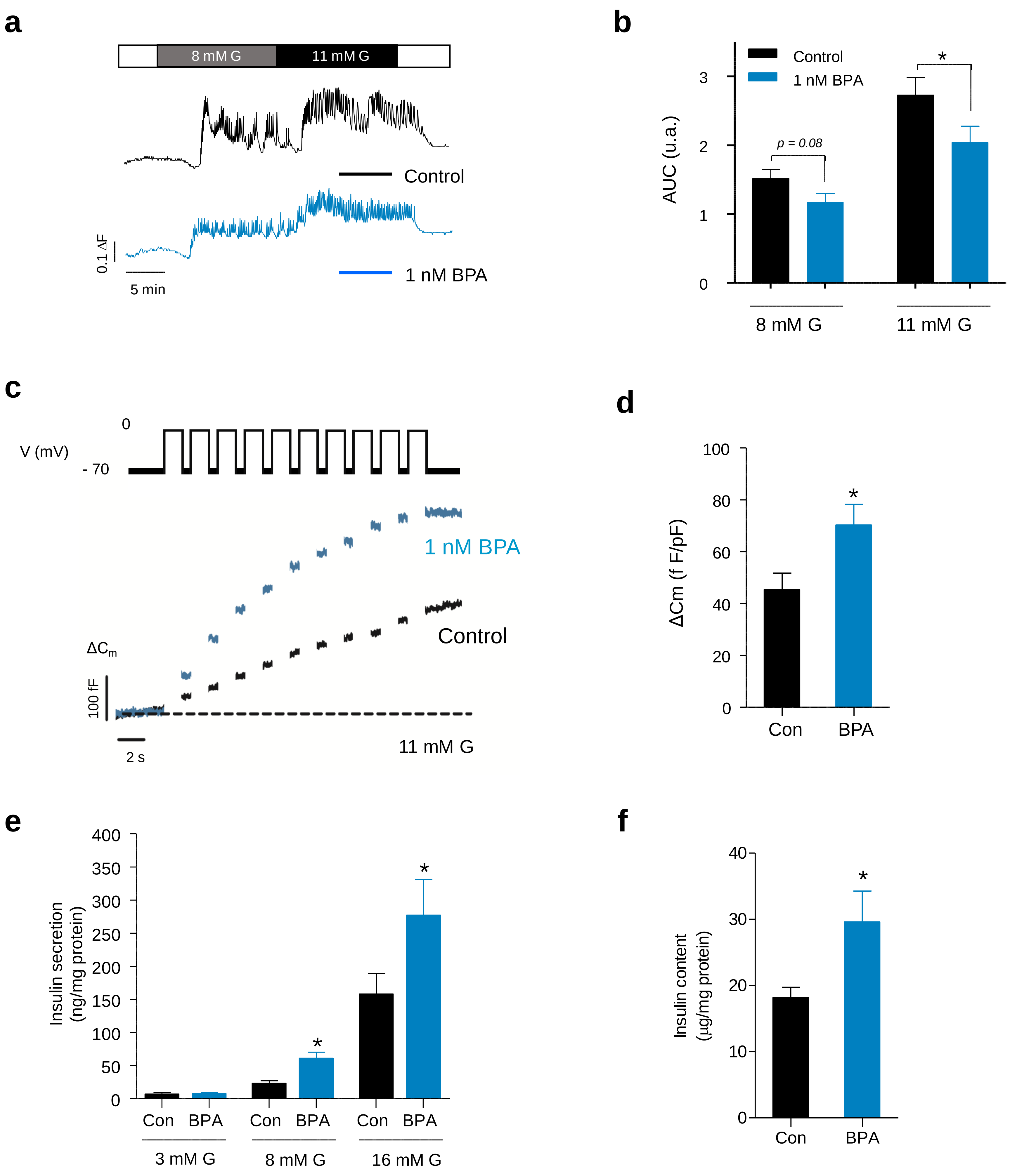

Figure S1. Effect of BPA on exocytosis, insulin secretion and Ca^{2+} entry at different glucose concentrations. (**a**) Representative superimposed recordings of fura-2 Ca^{2+} fluorescence in response to different glucose levels (3 mM (upper white boxes), 8 mM (upper grey box) and 11 mM (upper black box)) for whole islets left untreated (*Control*; black trace) or treated with 1 nM BPA (*1 nM BPA*; blue trace). (**b**) Average quantification of the area under the traces during the 10 minutes following the beginning of the fura-2 fluorescence changes in response to different glucose concentrations (8 and 11 mM) in the control (black bars, *n*=26 islets) or 1 nM BPA-treated (blue bars, *n*=29 islets) pancreatic islets. (**c**) Representative superimposed recordings of the membrane capacitance increase (lower panel) in response to depolarizing voltage steps (-70 to 0 mV, 500 ms duration; upper panel) in isolated β- cells left untreated (*Control*; black trace) or treated with 1 nM BPA (*1 nM BPA*; blue trace) 11 mM glucose. (**d**) Quantification of the average increase in capacitance (normalized to the cell size in pF) at the 10th voltage pulse of the experiment shown in (**c**) (control: *n*=15 cells; 1 nM BPA: *n*=20 cells; obtained from 4 experiments). (**e**) Increase in the insulin secretion of the islets (normalized to the islet protein content in mg) at 3, 8 and 16 mM glucose in islets left untreated (Control; black bar) or treated with 1 nM BPA (*1 nM BPA*; blue bar) (n= 7-11 groups of 5 islets per condition from 8 animals). (**f**) Insulin content of islets (normalized to the islet protein content in mg) of islets left untreated (Control; black bar) or treated with 1 nM BPA, samples were collected after insulin secretion (*1 nM BPA*; blue bar) (n= 35-38 groups of 5 islets from 8 animals). Data obtained represent the mean ± s.e.m. Measurements in (**b)** were obtained from cultured pancreatic islets from 6 independent experiments. Student's *t*-test: *P<0.05.

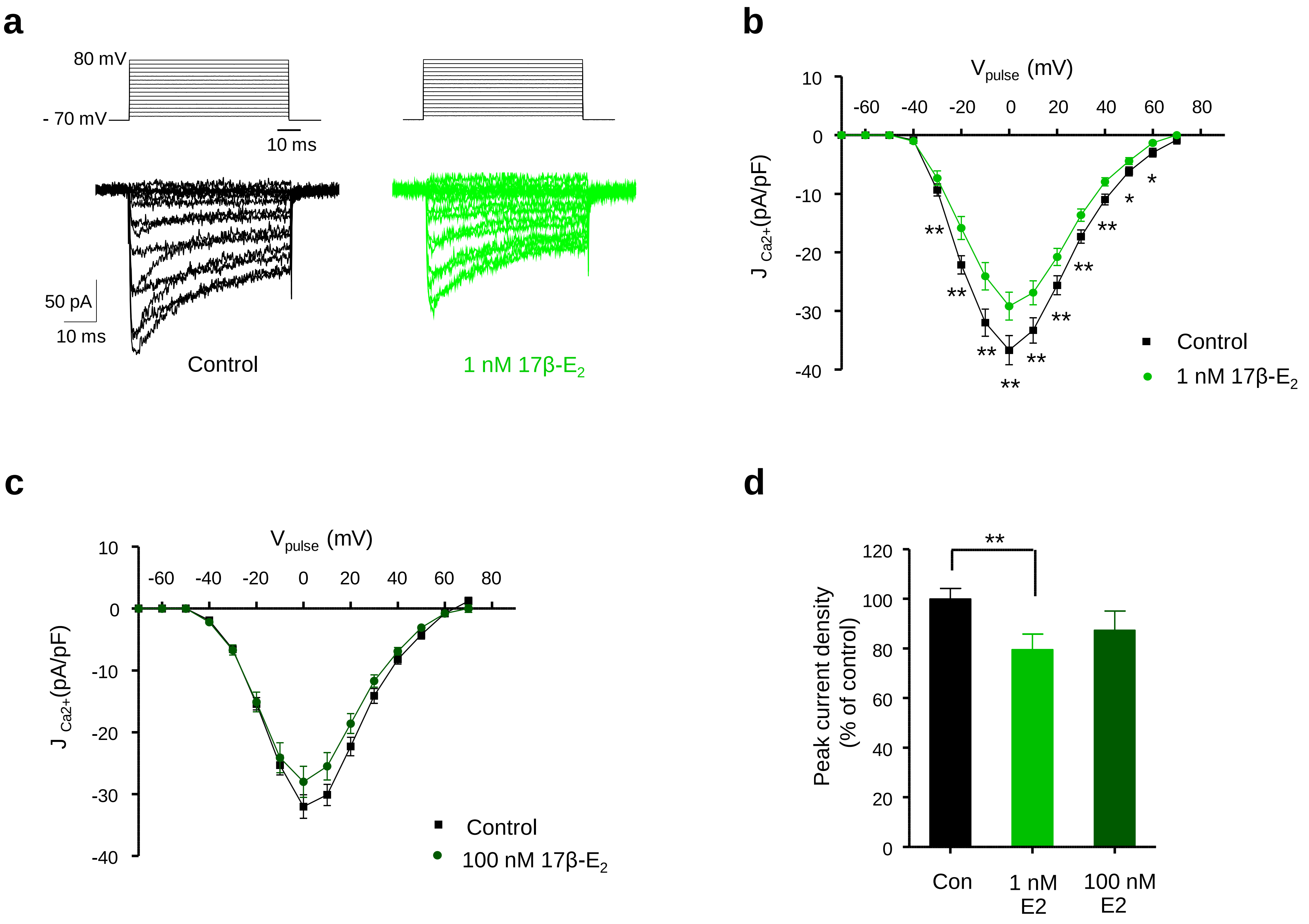

Figure S2. 17β-oestradiol inhibits the Ca^{2+} currents in mouse pancreatic β-cells. (**a**) Representative recordings of the Ca^{2+} currents (lower panels) in response to depolarizing voltage pulses (-60 mV to +80 mV from a holding potential of -70 mV, 50 ms duration; upper panels) in isolated β-cells left untreated (*Control;* left panels, black traces) or treated with 1 nM 17β-oestradiol (*1 nM 17 β-*E_2; right panels, green). (**b**) Average relationship between Ca^{2+} current density (J, Ca^{2+} currents normalized to the cell size in pF) and the voltage of the pulses in cells left untreated (*Control*; black squares, *n* = 16) or treated with 1 nM 17β-oestradiol (*1 nM 17 β-*E_2; green circles, *n* = 16). (**c**) The same experiment as in (**a-b)** but performed in β-cells exposed to 100 nM 17β-oestradiol (*Control*; black squares, *n*=15; *1 nM 17 β-*E_2; dark green circles, n = 17). (**d**) Average normalized values of current density evoked at 0 mV from the relationships shown in (**b)** and (**c**). Data obtained are represented as the mean ± s.e.m. Student's *t*-test: **P*<0.05; ***P*<0.01.

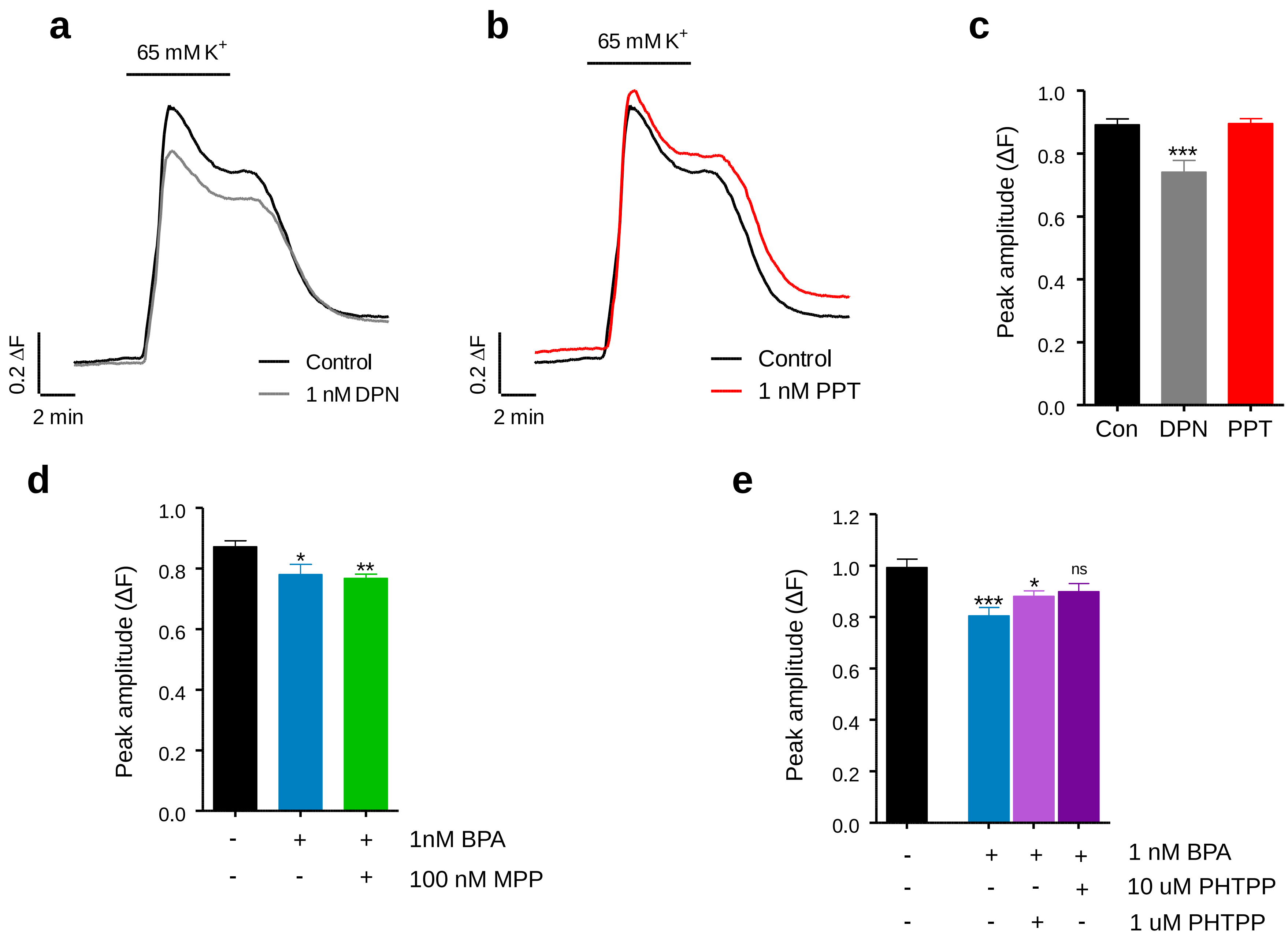

Figure S3. Inhibition of Ca^{2+} signalling by low doses of BPA involves oestrogen receptor β. (**a**) Representative superimposed recordings of fura-2 Ca^{2+} fluorescence in response to the extracellular application of 65 mM KCl in the presence of 100 µM diazoxide in isolated islet cells left untreated (*Control*; black trace) or treated with 1 nM DPN (*1 nM DPN*; grey trace). (**b**) As in (**a**), except isolated islet cells were left untreated (*Control*; black trace) or treated with 1 nM PPT (*1 nM PPT*; red trace). Note the lack of effect of PPT. (**c**) Average amplitude of fluorescence change measured in isolated islet cells from the experiments shown in (**a**) and (**b**) (*Control*; black bar, *n*=56; *1 nM DPN*; grey bar, *n*=50; *1 nM PPT*; red bar, *n*=63). (**d**) Average fura-2 Ca^{2+} fluorescence response to 65 mM KCl measured at the peak in isolated islet cells left untreated (*Control*; black bar, *n*=27), or treated with 1 nM BPA (*1 nM BPA*; blue bar, *n*=35) or 1 nM BPA + 100 nM MPP (*1 nM BPA + 100 nM MPP*; green bar, *n*=45). (e) Average fura-2 Ca^{2+} fluorescence response to 65 mM KCl measured at peak in isolated islet cells left untreated (*Control*; black bar, *n*=22), treated with 1 nM BPA (*1 nM BPA*; blue bar, *n*=24), or treated with 1 nM BPA + PHTPP as indicated below the figure (*1 µM PHTPP*; light purple bar, *n*=22; 10 µM PHTPP; dark purple bar, *n*=33). Data are represented as the mean ± s.e.m. One-way ANOVA followed by Dunnett´s post hoc test (vs. control group): ****P*<0.001; ***P*<0.01; **P*<0.05.

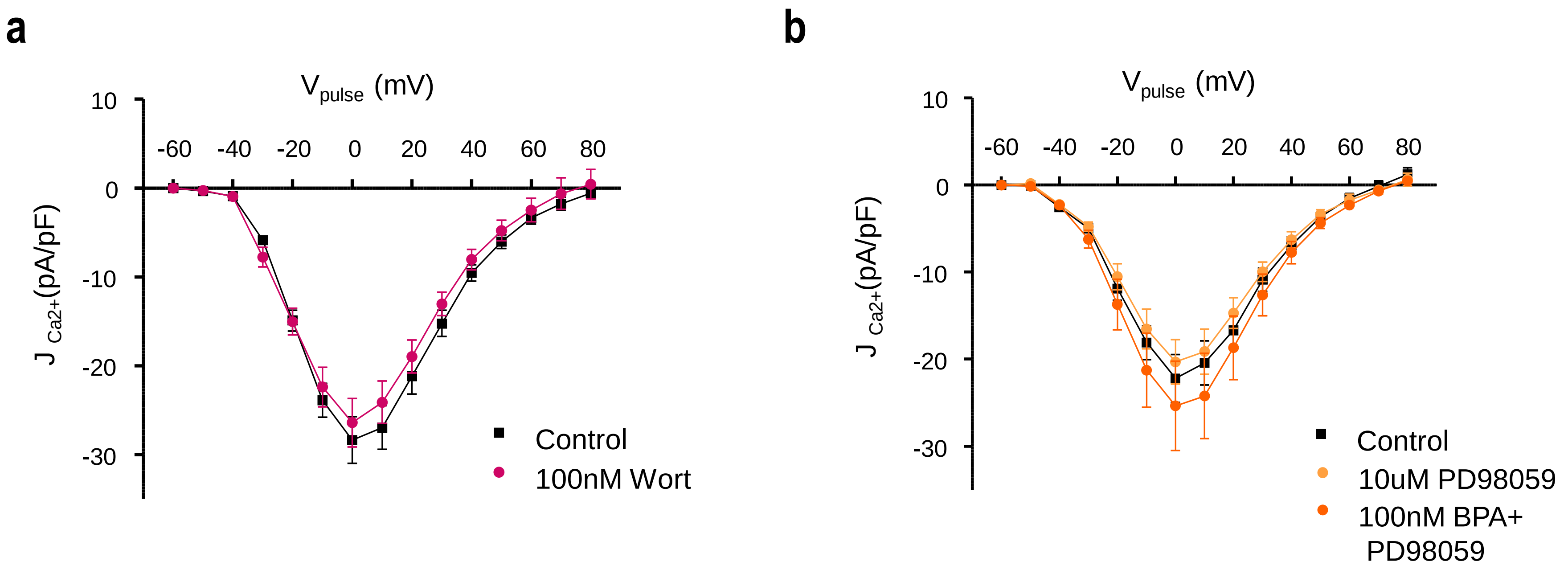

Figure S4. Potentiation of Ca^{2+} currents by high doses of BPA through oestrogen receptor α activation does not involve ERK1/2. (**a**) Average relationship between the Ca^{2+} current density and the voltage of the pulses in β-cells left untreated (*Control*; black squares, *n*=10) or treated with 100 nM wortmannin (*100 nM Wort*; magenta circles, *n*=14). (**b**) Average relationship between Ca^{2+} current density and the voltage of the pulses in β-cells left untreated (*Control*; black symbols, *n*=9), treated with 10 µM PD98059 (*10 µM PD98*; light orange circles, *n*=8) or 100 nM BPA + 10 µM PD98059 plus 100 nM BPA (*100 nM BPA + PD98*; dark orange circles, *n*=9). Data are represented as the mean ± s.e.m.
